# Towards isolated attosecond electron bunches using ultrashort-pulse laser-solid interactions

**DOI:** 10.1038/s41598-020-75418-6

**Published:** 2020-10-27

**Authors:** Jinpu Lin, Thomas Batson, John Nees, Alexander G. R. Thomas, Karl Krushelnick

**Affiliations:** Center for Ultrafast Optical Science, University of Michigan, Ann Arbor, MI 48109 USA

**Keywords:** Laser-produced plasmas, High-harmonic generation

## Abstract

We investigate MeV-level attosecond electron bunches from ultrashort-pulse laser-solid interactions through similarities between experimental and simulated electron energy spectra. We show measurements of the bunch duration and temporal structure from particle-in-cell simulations. The experimental observation of such bunches favors specular reflection direction when focusing the laser pulse onto a subwavelength boundary of thick overdense plasmas at grazing incidence. Particle-in-cell simulation further reveals that the attosecond duration is a result of ultra-thin ($\sim$tenth of a micron) gaps of zero electromagnetic energy density in the modulated reflected radiation, while the bunching (locally peaked electron concentration) comes from the highly-directional electron angular distribution acquired by the electrons in a grazing incidence setup. To isolate a single electron bunch, we perform simulations using 1-cycle laser pulses and analyze the effect of carrier-envelop phase with particle tracking. The duration of the electron bunch can be further decreased by increasing the laser intensity and the focal spot size, while its direction can be changed by tuning the preplasma density gradient.

## Introduction

Driving high-intensity ultrashort laser pulses into overdense plasmas tends to produce electron beams with higher charge number at lower peak energy compared to those from underdense plasmas in laser wakefield acceleration. This interaction between relativistic laser pulses and solids has been studied in the past decades in different regimes of laser pulse characteristics and plasma conditions. When the pulse duration is ultrashort ($\sim$ 30fs) and the plasma density scale-length is short compared to laser wavelength ($L_s=n_e\cdot (\frac{dn}{de})^{-1}<0.1\lambda$), the incident radiation drives a periodic motion of the critical surface to stretch and compress the light reflected off the surface within each cycle. This is known as the relativistic oscillating mirror model, which leads to the generation of attosecond light sources through high-order harmonic generation (HHG)^1–5^ as well as ejection of electrons from the surface. The reflected laser field then accelerates these electrons to relativistic energy through the vacuum laser acceleration (VLA) mechanism^6,7^, usually resulting in a ring-shaped electron beam structure^8,9^. If the plasma scale-length is longer than the laser wavelength, the interaction mainly happens near the critical density and the acceleration mechanism becomes extremely complex, including ponderomotive acceleration^10^, surface quasistatic fields^11^ and direct laser acceleration. Varying the scale-length from $<0.1\lambda$ to $\sim 5.5\lambda$, an optimal condition for generating quasi-monoenergetic electrons is found at $L_s=0.5\lambda$ using relativistic high repetition rate laser system with abundant statistics^12^. Scaling laws of electron temperature have also been studied^13^. A theory for the electron acceleration mechanism in this regime is standing-wave acceleration. Superposition of the incident half and the reflected half of the laser pulse results in a standing wave pattern, and the modulated electric and magnetic fields in the standing wave can launch electrons in the forward and backward directions^14–16^. Going to even shorter pulse duration matching the plasma wavelength ($t=\lambda _p/2c$), wakefields can be excited^17^. To have wakefield acceleration happen in laser-solid interaction, a few-cycle pulse driver ($\tau \sim T_0/2cos\theta$) is required^17^ so that large-amplitude plasma waves can be generated along the density gradient up to the effective turning point $n_{cr}cos^2\theta$.

Most of the reported laser-solid experiments were set up in normal^14,18,19^ or oblique^8,9,12,20^ ($\sim 45^{\circ }$) incidence. However, Naumova^21^ predicted the existence of attosecond electron bunches in a grazing incidence setup. Since then there have been extensive theoretical predictions and simulations for attosecond electron bunches using various geometries, such as droplet target^22,23^, nanofilm^24^ and transversely-thin slice target^25^, no experimental indication has been reported yet. In this work, we confirm that generating attosecond electron bunches favors a larger angle of incidence (measured from target normal) in both experiments and simulations. We utilize Particle-In-Cell (PIC) simulation and particle tracking to investigate the formation mechanism of these attosecond electron bunches. Furthermore, we generate isolated attosecond electron bunches using single-cycle laser pulses in PIC simulations. We study some key parameters that govern this process, such as preplasma density scale length, laser intensity, carrier-envelope-phase (CEP) and focal-spot size.

## Results

**Figure 1:**
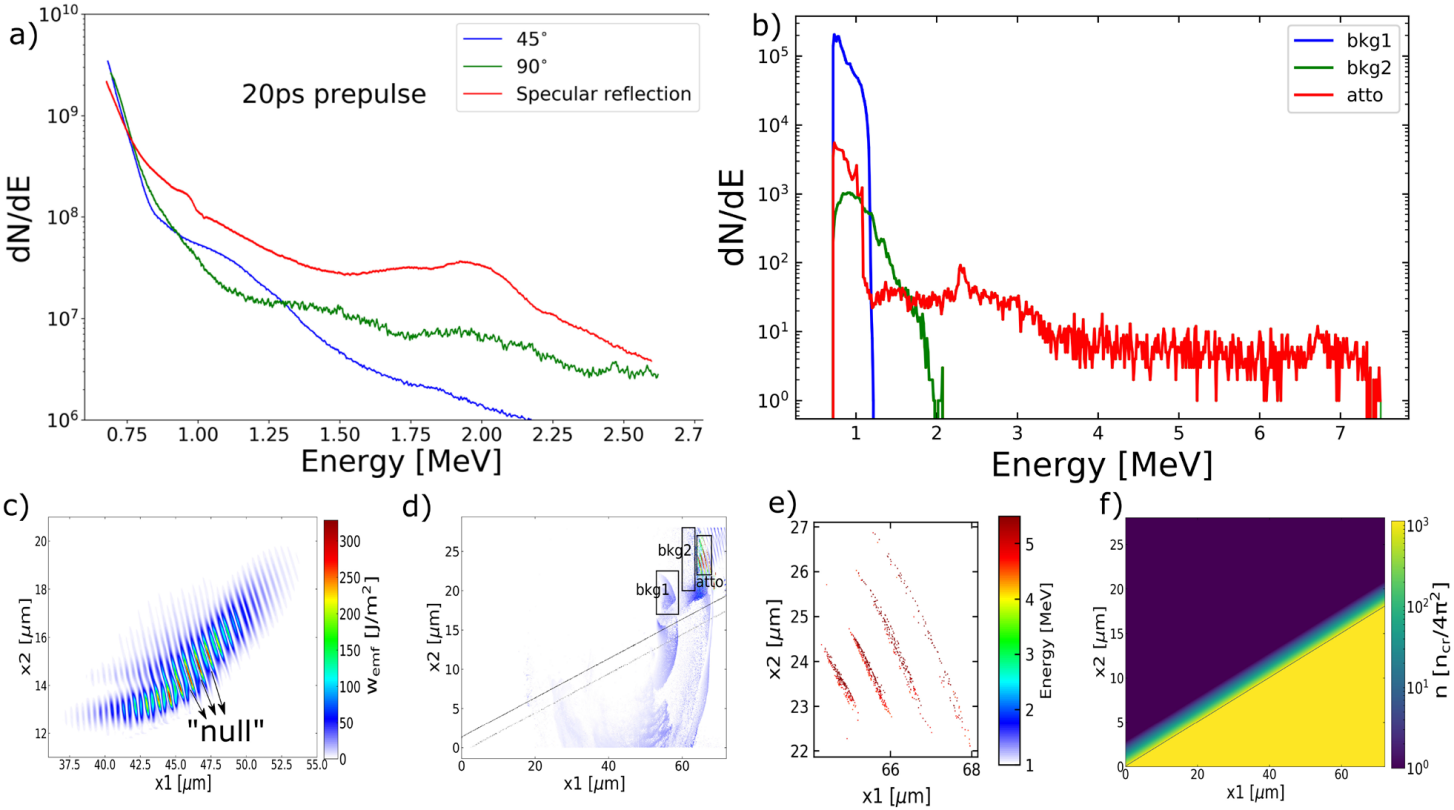
Electron energy spectra from experiments (**a**) and simulations (**b**) in grazing incidence setup. $45^{\circ }$, $90^{\circ }$ and specular reflection in (**a**) correspond to position 1, 2 and 3 labeled in Fig. 8c, respectively. (**b**): energy spectra of electrons labeled in (**d**). The bin size of the spectra is 72 bins/MeV. Both energy spectra in (**a**) and (**b**) have dn/dE in arbitrary units. (**c**): Electromagnetic energy density in the region of the reflected pulse at time t = 210 fs when peak laser intensity interacts with the critical surface. (**d**): Total energy of particles in the simulation box beyond 1 MeV at time t = 300 fs before the attosecond electron bunches leave the simulation box. (**e**): Particle energy of the attosecond electrons. It is the zoom-in of the square region “atto” in (**d**) with cutoff energy at 4 MeV. (**f**) Initial charge density in unit $n_0=n_{cr}/4\pi ^2$, where the scale-length is $L_s=0.5\lambda$.

Figure 1 demonstrates the similarities between the experimental and simulated electron energy spectra and the bunch duration measurement in simulation. The experiment was performed in a grazing incidence setup using a 20 ps prepulse and the measured electron energy spectra are shown in Fig. 1a. The experimental setup is shown in Fig. 8 under the Method section. All spectra have been calibrated using published image plate efficiency^26^. Figure 1b-f are from PIC simulations, which were also conducted in grazing incidence geometry with preplasma density scale-length matches the experimental prepulse. This conversion from prepulse to preplasma scale-length is performed in the 1D hydrodynamic code (HYADES^27^) assuming isothermal expansion.

Comparing the experiment and simulation results, we notice a “bump” feature in the red curves: near 2 MeV in Fig. 1a and left to 3 MeV in Fig. 1b. This feature does not show up in other curves in blue or green. In the simulations, the “bump” component of the electron spectrum is always observed along the target surface and was always associated with the train of ultra-short duration bunches of energetic electrons. Since the red spectra in Fig. 1b is taken from the attosecond electrons (bunch duration measured to be $\sim \lambda /8$ in Fig. 1e), it suggests the existence of attosecond electron bunches in the red spectra in the experiment. The propagation direction of the attosecond electron bunches is found to be in the specular reflection direction through particle tracking, matching the experimental observation.

**Figure 2:**
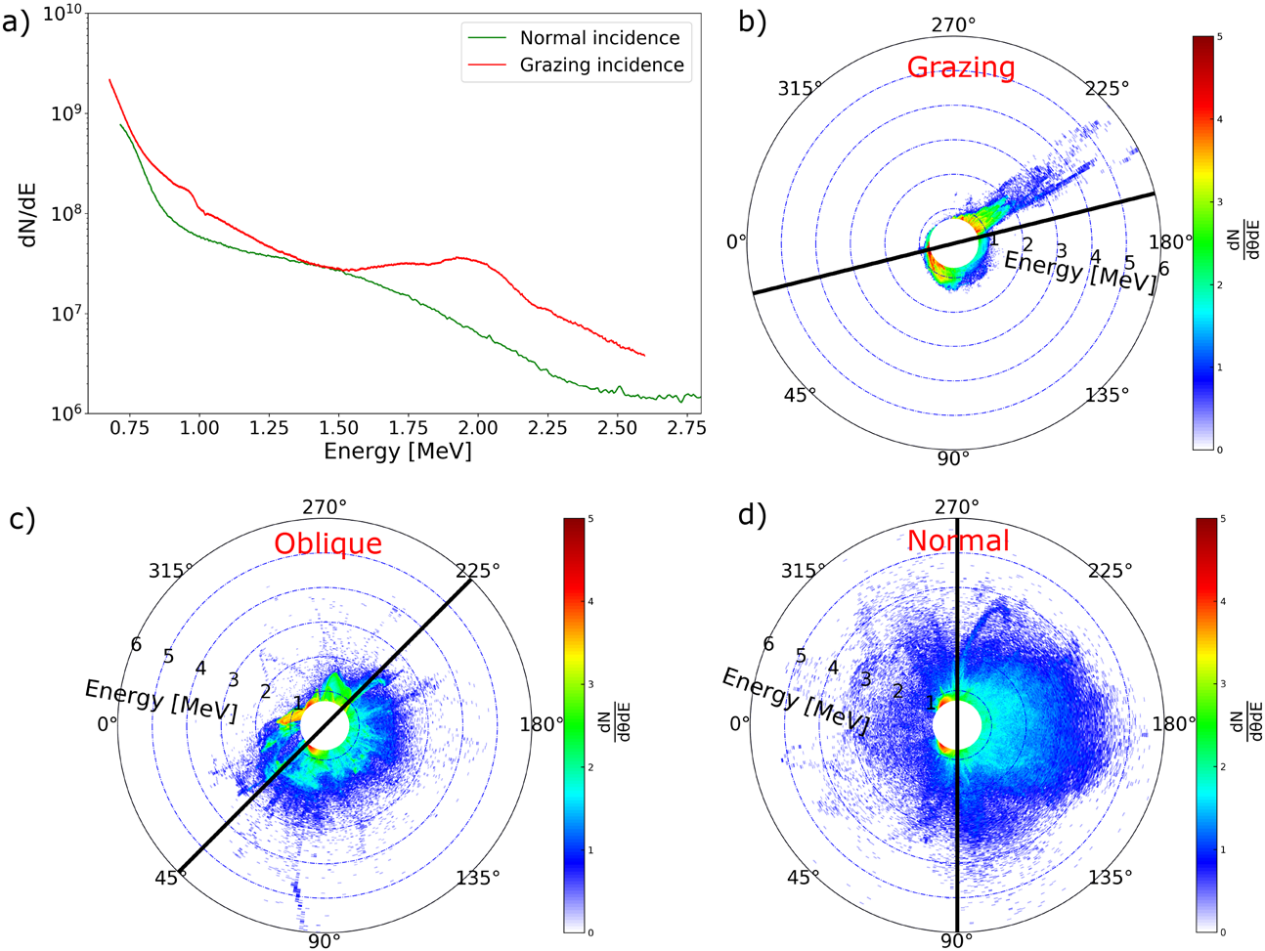
(**a**): Experimental electron energy spectra from normal incidence and grazing incidence cases using a 20 ps prepulse. (**b**–**d**): Simulated angular energy distribution of electrons for grazing, oblique and normal incidence using scale-length $L_s=0.5\lambda$. The laser pulse comes into the simulation box from the left ($0^{\circ }$) and the target lies along the black line. The snapshots were taken when peak laser intensity interacts with the critical surface in each case.

While Fig. 1 presents the direction of the emitted attosecond electron bunches, Fig. 2 investigates different incident angles of the laser pulses. The unique “bump” feature was observed only in the grazing incidence case but not in the normal incidence case, as is shown in Fig. 2a. To understand it, we ran simulations at different angles of incidence. Figure 2b–d shows the angular distribution of all electrons when peak laser intensity interacts with the critical surface. This is also the time when the ultra-thin “null” in the electromagnetic energy density is formed in Fig. 1c. Due to the self-intersection of electron trajectories, electron concentration is abruptly peaked^21^ to initialize the bunching process. Figure 2 shows that electrons are more spreading out in (c) and (d) than in (b), confirming that the bunching process favors grazing incidence.

It is worth pointing out that these observations are consistent with respect to both experiments and simulations. From the 41 data shots we took at different geometries, 25 were taken at the grazing incidence geometry that encourages attosecond electron bunches where the laser pulse was at grazing incidence and the spectrometer was positioned in the laser reflection direction. We observed the “bump” feature in 12 of the 25 spectra. We observed no spectra with this feature in the other 16 shots taken at different geometries. Energetic attosecond electron bunches with noticeable coherence were also observed consistently in simulations as we scanned the preplasma electron density scale-length from $0.1\lambda$ to $2\lambda$, and detailed results will be presented in the following section.

### Preplasma effects

**Figure 3:**
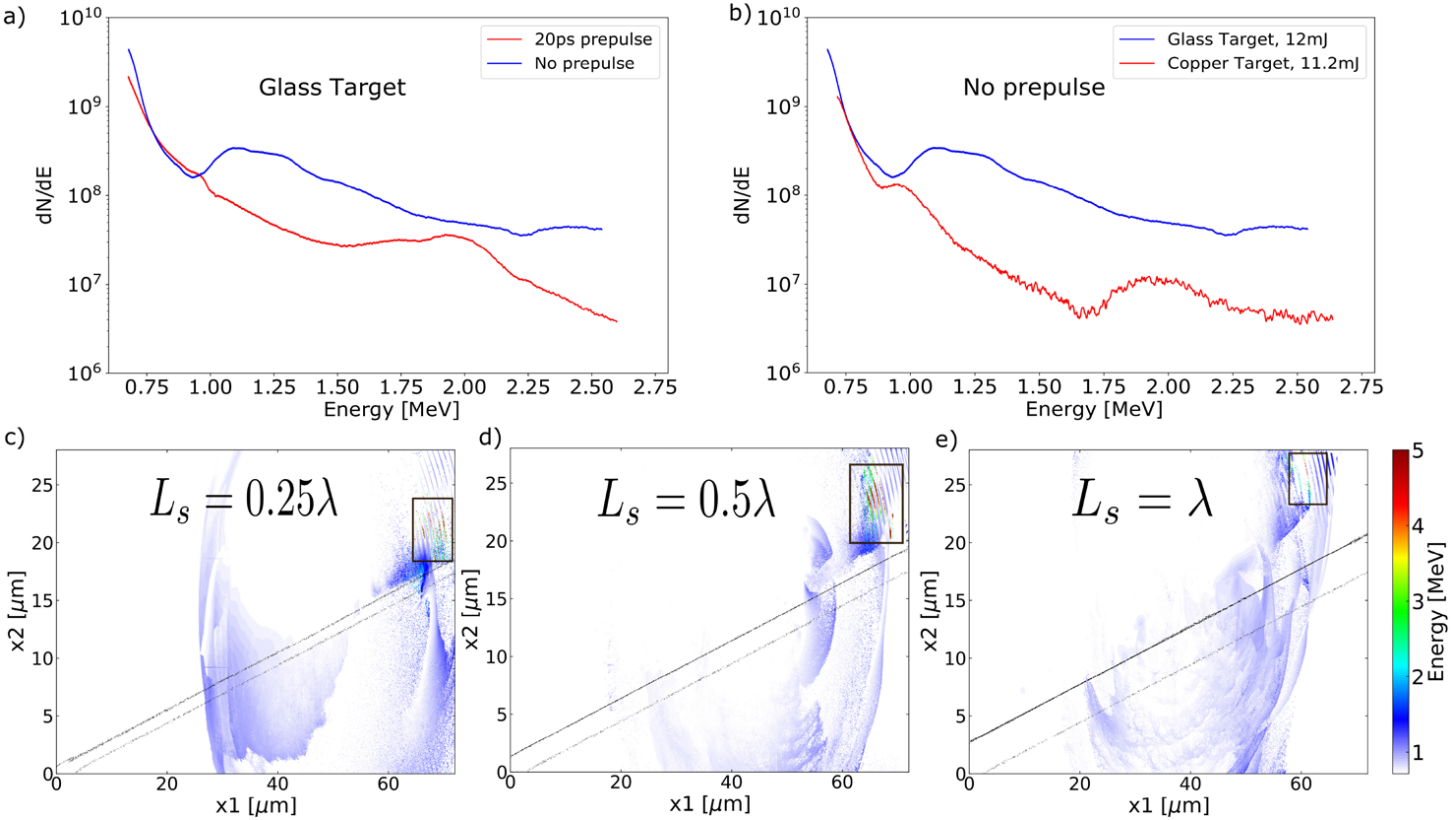
(**a**): Experimental electron energy spectra with and without an external 20 ps prepulse using glass target. (**b**): Experimental electron energy spectra using glass and copper target without external prepulse. (**c**)–(**e**): Simulated spatial distribution of energetic electrons using preplasma scale-length $L_s=0.25\lambda (c),\; 0.5\lambda (d)\; and\; \lambda (e)$. Both experiments and simulations were performed at grazing incidence geometry.

Preplasma density profile can affect the energy and propagation direction of the attosecond electron bunches. Figure 3a shows that including an additional 20 ps prepulse moves the “bump” feature towards right on the spectra, suggesting bunching at higher energy. The same phenomenon is observed in Fig. 3b as copper has a lower ionization threshold and thus more developed preplasma profile with longer scale-length when the main pulse arrives. To explore this effect in parameter space, we tune the preplasma density scale-length from $0.1\lambda$ to $2\lambda$ in simulation. Energetic attosecond electron bunches with noticeable coherence were observed in moderate scale-length cases, as is highlighted in Fig. 3c–e, but not in the two extreme cases where $L_s=0.1\lambda$ or $2\lambda$. Note that $L_s=0.5\lambda$ resulted in the most energetic attosecond electron bunches, matching the optimal condition for producing quasi-monoenergetic electron beams at $a_0\sim 2$ in previous experiments^12^. Besides, tuning the preplasma profile affects the bunch propagation direction. As the scale-length increases, the emission angle of the bunches become smaller measured from the target normal. This is due to the critical surface is moving away from the solid surface, so as the generated electron bunches.

### Carrier-envelope phase

**Figure 4:**
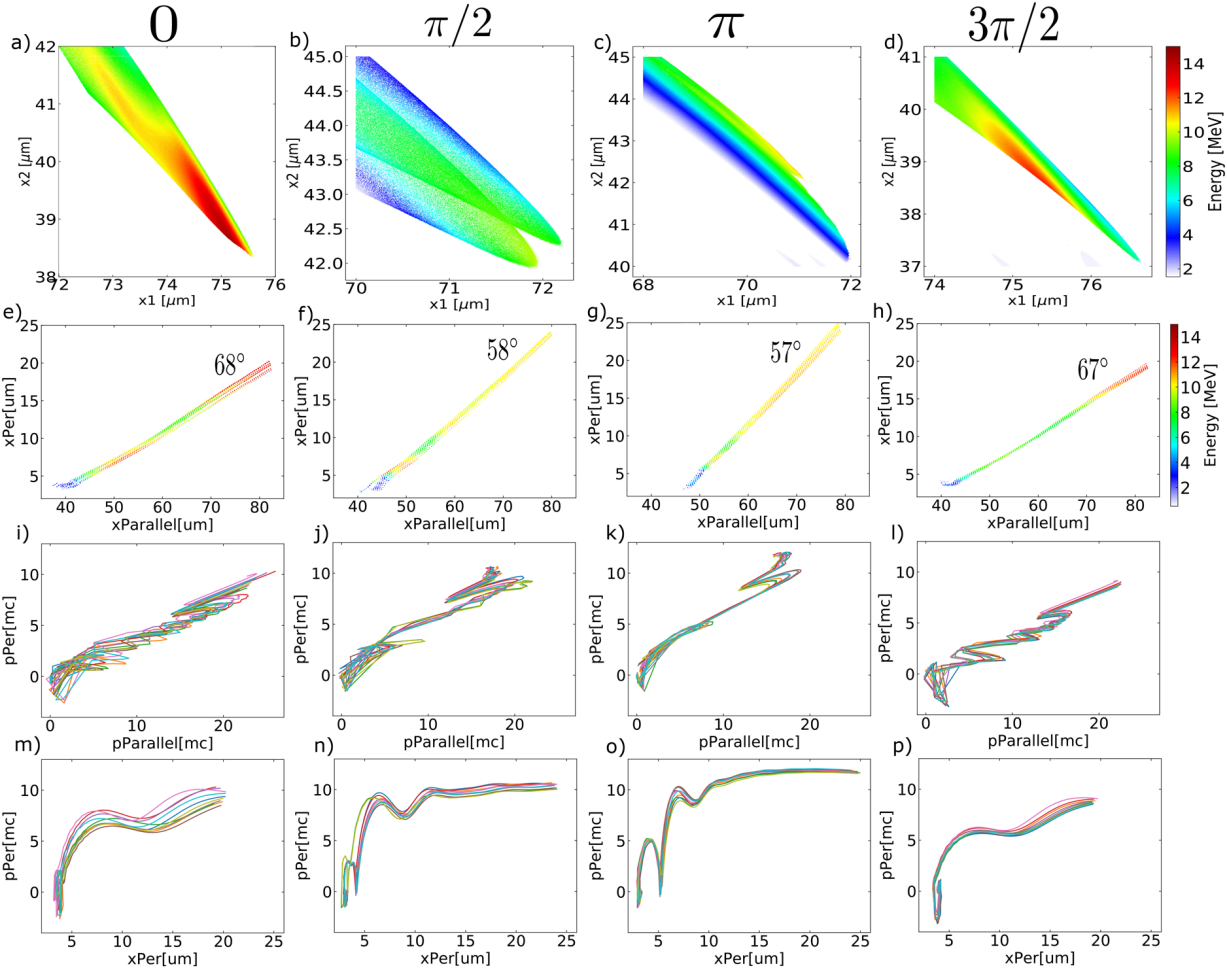
Single attosecond electron bunch and particle tracking using $CEP\;=\;0,\;\pi /2,\;\pi \;and\;3\pi /2$. (**a**)–(**d**): Spatial energy distribution of bunched electrons at the last time-step. (**e**)–(**h**): Trajectory of the attosecond electrons with emission angle labeled. (**i**)–(**l**): Perpendicular momentum vs. parallel momentum, colors representing different particles. (**m**)–(**p**): Perpendicular momentum change in the perpendicular axis.

To produce isolated attosecond electron bunches, we drove the interactions with single-cycle Gaussian pulses in the PIC simulation, keeping the above grazing incidence geometry but at higher normalized vector potential $a_0=10$. This is to produce electron bunches at higher energy and more separable from the background while keeping a comparable driving laser pulse energy. It has been shown that CEP plays a role in the electron acceleration process in LWFA^28^ and in nanoplasma acceleration^29^. Figure 4 demonstrates the generated single bunches using various carrier-envelope phases. It is observed that changing the CEP can dramatically affect the energy and shape of the electron bunch. It can also change the direction of the generated bunch slightly, but not as effective as changing the preplasma density profile. To better understand the effect of CEP on these electrons, we select ten macroparticles in the high energy part in Fig. 4a–d and track their trajectory from birth. The tracking results are shown in Fig. 4e–p in rotated axis perpendicular/parallel to the target surface. Electrons in the $CEP\;=\;\pi /2\;or\;\pi$ case in Fig. 4j,k experience a momentum loss in the final acceleration stage. On the other side, electrons in the $CEP\;=\;0\;or\;3\pi /2$ case in Fig. 4i,l experience the momentum loss in earlier stages but find the correct phase with respect to the reflected laser field in later time to gain energy. This is in line with the difference in the final energy of the accelerated electrons in Fig. 4a–d,e–h. Figure 4e–h also provide the emission angle of the bunched attosecond electrons: $\sim 68^{\circ }$ in $CEP\;=\;0\;or\;3\pi /2$ case and $\sim 58^{\circ }$ in $CEP\;=\;\pi /2\;or\;\pi$ case. The emission angle is defined as the angle between the electron bunch propagation trajectory and the target normal direction. It is worth noting that electrons have negative initial perpendicular momentum in Fig. 4m–o, moving towards the high-density target before the ejection. Electrons have positive initial perpendicular momentum and travel away from the target can not eject through the electromagnetic energy density gap until pulled back towards the target, as is shown in Fig. 4p. However, despite losing the initial momentum, these electrons are not slowed down once they get ejected, as opposed to the momentum decrease of electrons in Fig. 4n,o at $x_{per}\sim 5\mu m$. Controlling CEP offers the ability to inject electrons with various initial phases, which is important in the later stage of phase-change and acceleration.

### Focal-spot size

**Figure 5:**
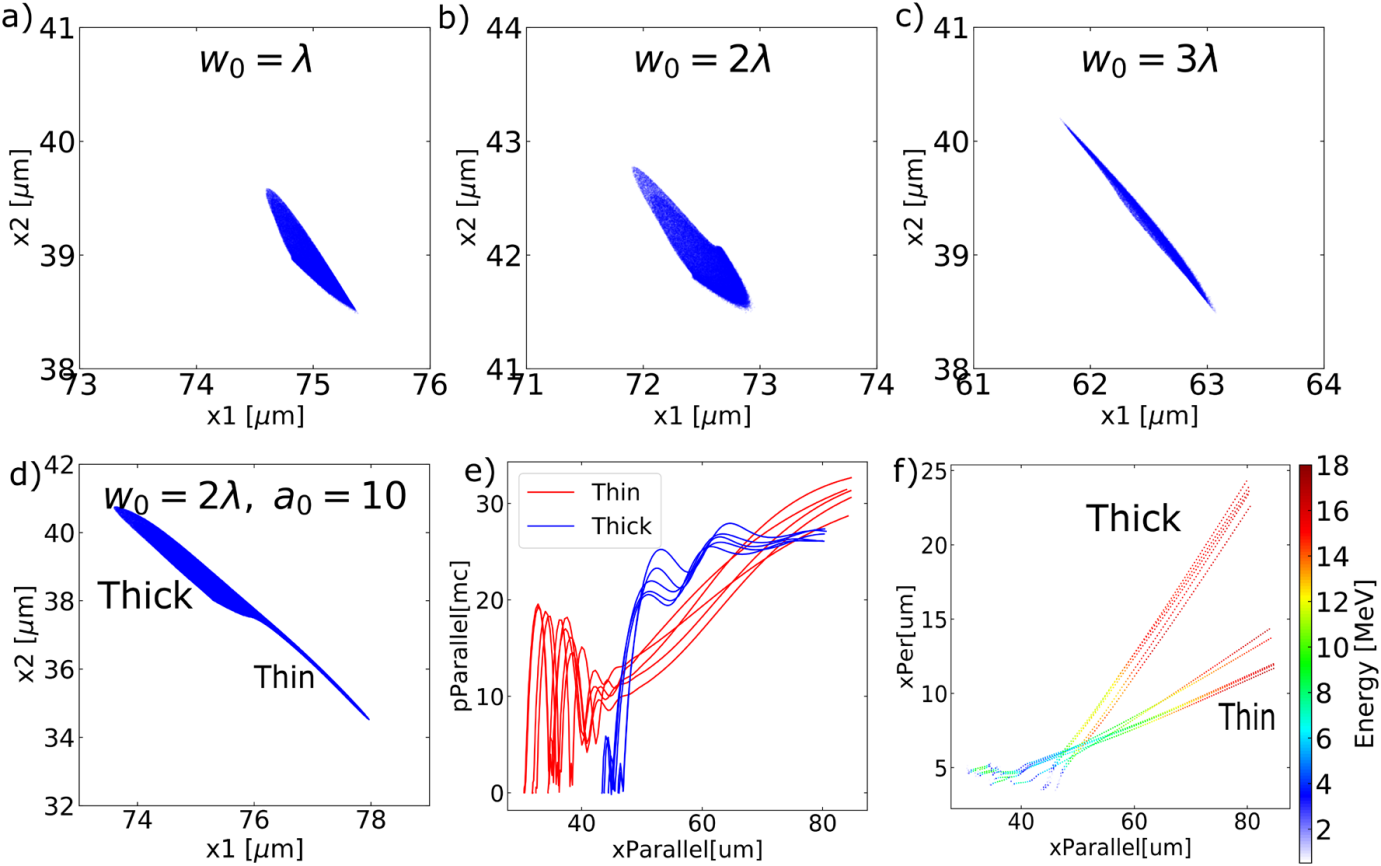
Spatial profiles of attosecond electron bunch generated from different focal spot size, keeping the same pulse energy (**a**–**c**) or the same $a_0$ (**a**,**d**). Cutoff energy in (**a**)–(**d**) is set at the high energy edge on each spectra: 13 MeV, 9 MeV, 5 MeV and 18 MeV, respectively. (**e**) and (**f**) are the particle tracking results of the thick and thin part of the bunch in (**d**).

Focusing the laser pulse to a larger focal spot can reduce the attosecond electron bunch duration. Figure 5a–c keeps the same pulse energy and varies the focal beam waist, achieving a thinner bunch at $w_0=3\lambda$. The number of electrons in these bunches are in ratio of 4:3:2. It is as expected that larger focal spot produces shorter bunches. In our grazing incidence setup using tight focus, the rays cover a range of angles $\alpha \sim 1/(2\cdot f/\#)$. When focusing the beam to a small focal spot $w_0=\lambda$, this angle is around $20^{\circ }$. Given the incident angle of the central ray is $76^{\circ }$, the rays cover from $56^{\circ }$ to grazing. When the focal spot size is increased, this angle alpha decreases, i.e., $\alpha ^{'} < \alpha$ and the rays cover from $\beta$ ($\beta =76^{\circ }-\alpha ^{'}>56^{\circ }$) to grazing. Hence overall the rays are incident at larger angles. Note that large incident angles are preferred to produce attosecond electron bunches, as is shown in Fig. 2, and larger incident angles would lead to shorter electron bunch duration, as is shown in Eq. (5). A sketch of the ray angles in the focal spot geometry can be found as Supplementary Fig. S1 online.

If we keep $a_0=10$ and increase the focal beam waist, an extra thin bunch is observed in addition to the thick bunch in Fig. 5d. Particle tracking reveals that the thin bunch originates from earlier injection at $x_{parallel}\sim 30\mu m$ in Fig. 5f, while the thick part (as well as the bunch from the $w_0=\lambda$ case in Fig. 4e) originate from $x_{parallel}>40\mu m$. Tracking the evolution of electron momentum along the target surface direction in Fig. 5e, thin-bunch electrons (in red) oscillate within the laser field over a few cycles as it interacts with the dense plasma. They then get accelerated without the momentum loss that the thick-bunch electrons (in blue) experience, expected to reach even higher energy as they propagate.

### Energy-wise bunch characteristics

**Figure 6:**
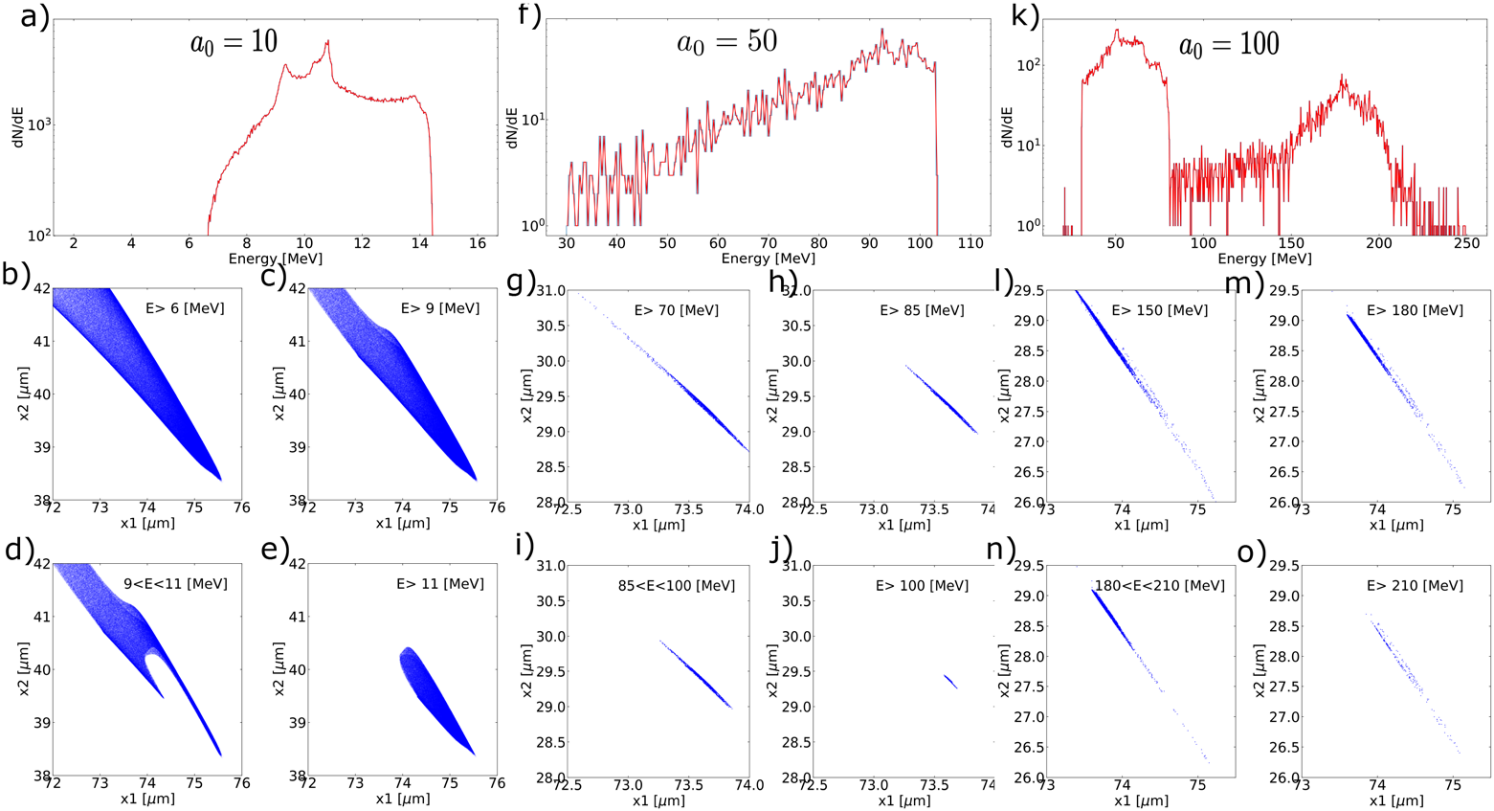
Energy spectra and energy-wise bunch duration using $a_0=10$ in (**a**)–(**e**), using $a_0=50$ in (**f**)–(**j**) and using $a_0=100$ in (**k**)–(**o**).

Duration of the isolated attosecond electron bunch and the electron concentration have been measured against energy. Figure 6a–e measure the bunch in Fig. 4a ($a_0=10$, CEP=0, $w_0=\lambda$). Figure 6f–o measure the bunch using all the same parameters but $a_0=50$ and $a_0=100$. Energy spectra in Fig. 6a,f,k again show the “bump” feature, and the shape of these most concentrated electrons are shown in Fig. 6d,i,n, respectively. The low energy part in the $a_0=100$ case represents background electrons that spatially overlap with the bunch at this time snap, which goes away as the bunch propagates. Movies that track the electron bunches can be found as Supplementary Video S1 online. Figure 6b–e,g–j,l–o present the spatial profile of the electron bunch at different cutoff energy, labeled respectively. Comparing them indicates that going to higher $a_0$ leads to a thinner bunch at higher energy. Note that the optimal scale-length for energetic electron bunch decreases with $a_0$. We have scanned multiple scale-lengths and present $L_s=0.25\lambda$ for the $a_0=50$ case and $L_s=0.1\lambda$ for $a_0=100$. It is worth pointing out that these electron bunches hardly see any radiation reaction effects even when $a_0=100$. Classical radiation reaction effects becomes strong when the classical radiation reaction parameter $R_c\sim a_0\chi >0.01$ and dominate when $R_c>0.1$^30^, where $\chi$ is the relevant parameter for supercritical fields. To consider radiation reaction effects when $a_0=100$, one needs $\chi >0.01$. However, this is not available in our case even in the frame of the electrons because the beam energy is not high enough. The fact that the electron bunch is almost co-propagating with the reflected laser field makes $\chi$ even smaller as $\chi \sim \gamma |E|(1-\beta \cos {\theta })$, where $\theta$ is the angle between electron momentum and wave vector. In fact, these attosecond electron bunches produced under this geometry should see even weaker fields than the fields in the lab frame as $\theta \sim 10^{\circ }$ and $\cos {\theta }\sim 1$.

Another motivation to go to higher energy is to propagate the beam further as attosecond bunches before being dispersed to longer duration. The spatial dispersion between the fastest and slowest electron in a bunch is estimated using Eq. (1), where $\gamma$ is the Lorentz factor.

1 $$\begin{aligned} d L=0.5 *\left( \frac{1}{\gamma _{\text{ low }}^{2}}-\frac{1}{\gamma _{\text{ high }}^{2}}\right) \cdot L \end{aligned}$$

For few-MeV electron bunches as in Fig. 1e, the dispersion *dL*/*L* is calculated to be 4e-3. The duration between the fastest and slowest electrons would exceed attosecond levels (beyond 1fs) at 0.1mm away from the source. We have also characterized the dispersion of the high energy electron bunches in Fig. 6. The estimated dispersion *dL*/*L* is 4e-4, 1e-6 and 6e-7 for the bunch in Fig. 6e,j,o, respectively. In other words, if the bunch in Fig. 60 propagates for one meter in free space, the spacing between its front and back edge will be 0.6 $\mu m$. Its bunch duration can maintain below 1 fs until it propagates 0.45m. For direct measurements of the bunch duration, higher energy electrons with less dispersion are clearly favored. Such beams will also produce more optical transition radiation which will make them easier to characterize.

### Tilted laser pulses

**Figure 7:**
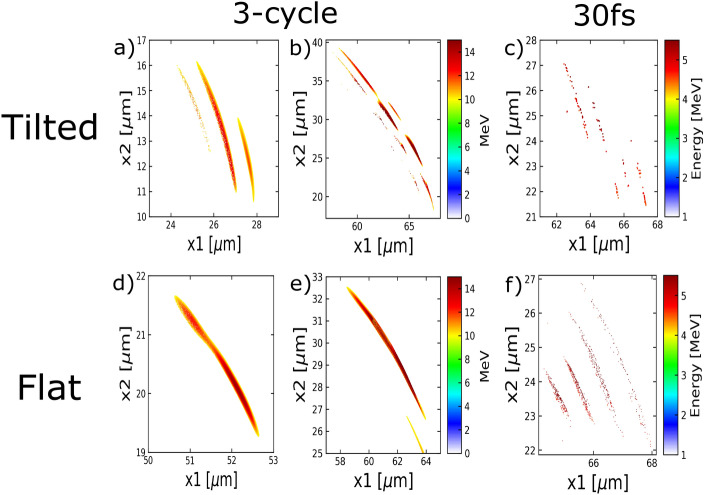
Attosecond electron bunches generated using tilted pulses in (**a**)–(**c**) versus flat pulses in (**d**)–(**f**).

Apart from using single-cycle laser pulses, another approach to obtain isolated attosecond electron bunches is using tilted laser pulses. The idea is to spatially separate the electron bunches by rotating the wave vector within a pulse. This concept of spatial-temporal couplings in ultrashort pulses was first explored theoretically by Akturk et al.^31^ and was experimented to produce isolated attosecond pulses by Wheeler et al.^32^. We show that this method also applies to the electron bunches as the tilted laser pulse interacts with solid-density plasmas. Figure 7a presents the spatial profile of energetic electrons (>10 MeV) produced by a tilted 3-cycle pulse, where a perpendicular spatial chirp $d\omega /dx_{2}$ is included at the focus. This can be achieved by introducing an angular dispersion in the laser beam, which allows different frequency components of the laser pulse disperse along the perpendicular axis ($x_{2}$) and leads to the spatial chirp. As a result, the laser cycle period ($T_{l}=c/\omega$) varies along the perpendicular axis. This is equivalent to a perpendicularly varying spacing between laser field cycles in the spatial domain, which leads to a rotating wave vector. An intuitive illustration can be found in Fig. 1 in Wheeler et al.’s work^32^. The three cycles of the laser pulse go to focus at three different angles and produce three separate bunches, as is shown in Fig. 7a. Compared to the central bunch generated during the second laser cycle, the first and third bunch are shorter in length as the interacting position varies over time and the phase of “null” EM energy density lasts longest amid the second cycle. Note that Fig. 7a shows the electron bunches shortly after the laser pulse interacts with the dense plasma, while Fig. 7b presents the profile of these electrons when they leave the simulation box. A movie that tracks the propagation of these three bunches in every half-cycle time-step can be found as Supplementary Video S2 online. We also apply the tilted wavefront concept to a 30 fs laser pulse, which is more readily accessible in high power laser facilities. The resulted energetic electrons (>4 MeV) are presented in Fig. 7c with observable separation as well. For reference, Fig. 7d–f show the energetic electron bunches from a flat wavefront without any spatial chirp using a 3-cycle pulse and a 30fs pulse, respectively. In conclusion, spatial-temporal couplings in ultrashort pulses can be inherited by the electron bunches through interaction with dense plasmas. However, maintaining these properties through propagation requires fine measures.

## Discussion

Attosecond electron bunches are a unique product of ultra-short laser-solid interactions at grazing incidence geometry: “attosecond” comes from the ultra-thin “null” in the electromagnetic energy density shown in Fig. 1c while “bunch” comes from the peak electron concentration shown in Fig. 2b. Electrons oscillating in the fields at the correct phase can eject away from the target through the “null”, which is the cause of the ultra-short duration of the bunched electrons. On the other side, the “bunch” is formed by the peaked electron density that these ejected electrons inherit^21^, which prefers a large laser incidence angle.

The electrons are accelerated in the reflected laser pulse after being ejected from the target surface. This process is sensitive to the phase of the laser pulse as well as the initial phase of the electrons. Consider the direct acceleration of a single electron in a plane wave^33^, it gains momentum through $p_{z}-p_{z 0}=\gamma -\gamma _{0}$ and $p_{\perp }-p_{\perp 0}=\mathbf {a}$ where $p_{z}$ is the longitudinal momentum and $p_{\perp }$ is the transverse momentum. Ejected electron usually starts with some non-zero momentum, as is shown in the particle tracking, and obtains longitudinal momentum by $\frac{a^{2}+2a\cdot p_{\perp 0}}{2\left( \gamma _{0}-p_{z 0}\right) }$. There is a more generalized theory that describes the energy gain in three-dimensional space with dephasing^34^. In addition to the single electron model, the collective behavior of the plasma is nontrivial. Depending on the direction of the incident electric field in a single-cycle pulse, electrons move towards or away from the pulse to compress or stretch it. The vector potential and the electric field of the reflected pulse are^35^:

2 $$\begin{aligned}&A_r=2\pi \int _{0}^{t^{\prime }} d \tau I_{y, e}(\tau )+I_{y, i} \xi _{r}, \;\;I_y=-e N_{0} V_{y} \end{aligned}$$

3 $$\begin{aligned}&E_r=\frac{2 \pi N_{0} e\left( c \kappa _{y}+e A_{y}(\xi )\right) \mathcal {E}}{m_{e}^{2} c^{4}+\left( c \kappa _{y}+e A_{y}(\xi )\right) ^{2}}+\frac{2 \pi N_{0} e}{c} V_{y} \end{aligned}$$

where $\xi$ is the initial phase of the laser pulse, $N_0$ is the plasma density and $V_y=c\sin (\theta )$ shifts an oblique incidence angle $\theta$ to normal incidence frame^35^. Knowing the vector potential from Eq. (2) we can determine the momentum gain $p_z$ in the modulated reflected fields:

4 $$\begin{aligned} p_{z}=p_{z 0}+\frac{a_r^{2}+2a_r\cdot p_{\perp 0}}{2\left( \sqrt{1+p_{z 0}^{2}+p_{\perp 0}^{2}}-p_{z 0}\right) }, \;\;\;a_r=e A_r / m_{e} c^{2} \end{aligned}$$

It gives an estimation of the electron energy in the bunches. The electron bunch duration can also be predicted by estimating the “null” thickness in the electromagnetic energy density. Setting $E^2+B^2=0$, the phase of the reflected wave becomes $\xi _{r}=\int (1-a(2\pi \xi /\lambda )cot(\theta ))d\xi$ , where $a(2\pi \xi /\lambda )$ is the vector potential of the incident wave. For energy density $0^+$ and $0^-$,

5 $$\begin{aligned} \Delta \xi _{r}\sim [a(2\pi \xi ^+/\lambda )-a(2\pi \xi ^-/\lambda )]cot(\theta ) \end{aligned}$$

where $\xi ^+$ and $\xi ^-$ are the phases which correspond to density $0^+$ and $0^-$. This agrees with the simulation and experimental results that a larger incident angle is preferred. It also indicates that to get shorter electron bunch duration, a slowly-varying vector potential is preferred in phase space, or equivalently longer laser wavelength. Further experimental studies are expected to verify this prediction.

It should also be pointed out that the carrier-envelope phase of single cycle pulses could affect the electric field strength directly. The intensity profile of a Gaussian pulse is:

6 $$\begin{aligned} I(t)=\epsilon cn \cdot \frac{1}{T} \int _{t-\frac{T}{2}}^{t+\frac{T}{2}} |E(t')|^{2} dt'=\epsilon cn E_{0}^{2} \cdot \frac{1}{T} \int _{t-\frac{T}{2}}^{t+\frac{T}{2}} e^{-(t' / \tau )^{2}} \cos ^{2}(\omega t'+\phi ) dt', \end{aligned}$$

In most cases when the pulse duration is much longer than one optical cycle, $t>T=2\pi /w_0$, we can apply the slowly varying envelope approximation (SVEA) and the intensity profile becomes: $I(t)=\epsilon cn E_{0}^{2}e^{-t^2/\tau ^2} \cdot \frac{1}{T} \int _{t-\frac{T}{2}}^{t+\frac{T}{L}} \cos ^{2}(\omega t'+\phi ) dt'=I_0e^{-t^2/\tau ^2}$, where $I_0=\frac{1}{2}\epsilon cn E_{0}^{2}$ is the peak intensity. However, SVEA is no longer valid when the pulse duration is comparable to one optical cycle. In our case with a single cycle pulse, we have to integrate both the CEP term and the exponential term in Eq. (6). The peak intensity scales with the electric field amplitude as $I_0\sim 0.441\epsilon cn E_{0}^{2}$ for $CEP=0\;or\;\pi$ and $I_0\sim 0.454\epsilon cn E_{0}^{2}$ for $CEP=\pi /2\;or\;3\pi /2$, which are different from the commonly used formula $I_0\sim 0.5\epsilon cn E_{0}^{2}$ under SVEA. Thus for a fixed laser intensity, the carrier electric field is slightly stronger in the $CEP=0\;or\;\pi$ cases. Note that this is not exactly the electric field that drives the plasmas: the absolute phase of the carrier wave slides underneath the envelope as the beam focuses, especially in single-cycle pulses. This is due to the Gouy phase shift generated by isodiffracting ultra-braodband pulses through the beam waist^36^. Besides the vacuum focus, plasmas act as another focusing lens as the ponderomotive force pushes the electrons outwards. The phase change due to the “plasma lens” is dependent on its thickness and refractive index, which are essentially determined by the preplasma density profile.

Experimental measurement of ultra-short electron bunch duration can be accessible by measuring characteristics of coherent optical transition radiation (COTR) produced by the electrons. When an electron bunch incidents on an interface between two media with different refractive index, coherent electromagnetic fields are radiated and the electron bunch duration can be inferred. For example, Lundh *et al.*^37^ measured the COTR from an electron beam generated in a colliding pulse injection regime, and compare the COTR spectrum to analytic Gaussian COTR spectra for different bunch duration to deduce the bunch duration is $\sim$ few fs. Besides, Sears *et al.*^38^ measured the duration of electrons from inverse free-electron-laser process to be $\sim 410$ attoseconds. While using overdense plasmas in a grazing incidence setup, electron bunch duration can potentially be shorter than a tenth of a femtosecond. Since shorter electron bunches produce transition radiation at shorter wavelengths, to measure the duration of such electron bunches requires diagnosing the COTR further in the x-ray regime. These attosecond electron bunches can potentially be applied to probe the temporal evolution of dynamic systems such as magnetic fields formation^39^ and attosecond electron microscopy and diffraction^40^. It would also be worth trying to drive the interactions with laser pulses that carry orbital angular momentum^41,42^ to further manipulate the electron bunch characteristics.

To sum up, we show the experimental electron energy spectra from driving 30 fs, 800 nm laser pulses at grazing incidence onto a 6 mm thick glass target. The experimental energy spectra match the spectra of attosecond electron bunches observed in simulations. The duration of the bunches are measured to be $\sim$tenth of a micron ($\sim$100 attoseconds) in simulations. Direct experimental measurement of the bunch duration was not performed, although it can potentially be achieved by measuring characteristics of COTR produced by the electrons into the x-ray regime. We find grazing incidence geometry is necessary to produce attosecond electron bunches in simulations and corresponding spectral features in experiments. We show that the generation of energetic attosecond electron bunches favors larger incident angle, higher pulse energy, larger focal spot size, and moderately sharp preplasma density profile. We obtain isolated attosecond electron bunches using single-cycle pulses. Controlling CEP offers the ability to inject electrons with various initial phase and to adjust the energy and shape of the bunch. Due to the Guoy phase shift, CEP is changing as the single-cycle pulses focus through the plasmas. Preplasma density profile governs the propagation direction of the bunch and fine-tuning of the direction is accessible by tuning CEP. Higher $a_0$ and larger focal spot size can result in even shorter bunch duration with less dispersion after propagation. When operating with much higher pulse energy, sharper preplasma profile is preferred to produce a cleaner bunch. Using tilted laser pulses can pass angular properties to electron bunches and spatially separate them. With a simplified analytic model we predict the momentum gain of electrons and we predict that shorter electron bunch duration scales with larger incident angle and longer laser wavelength.

## Methods

**Figure 8:**
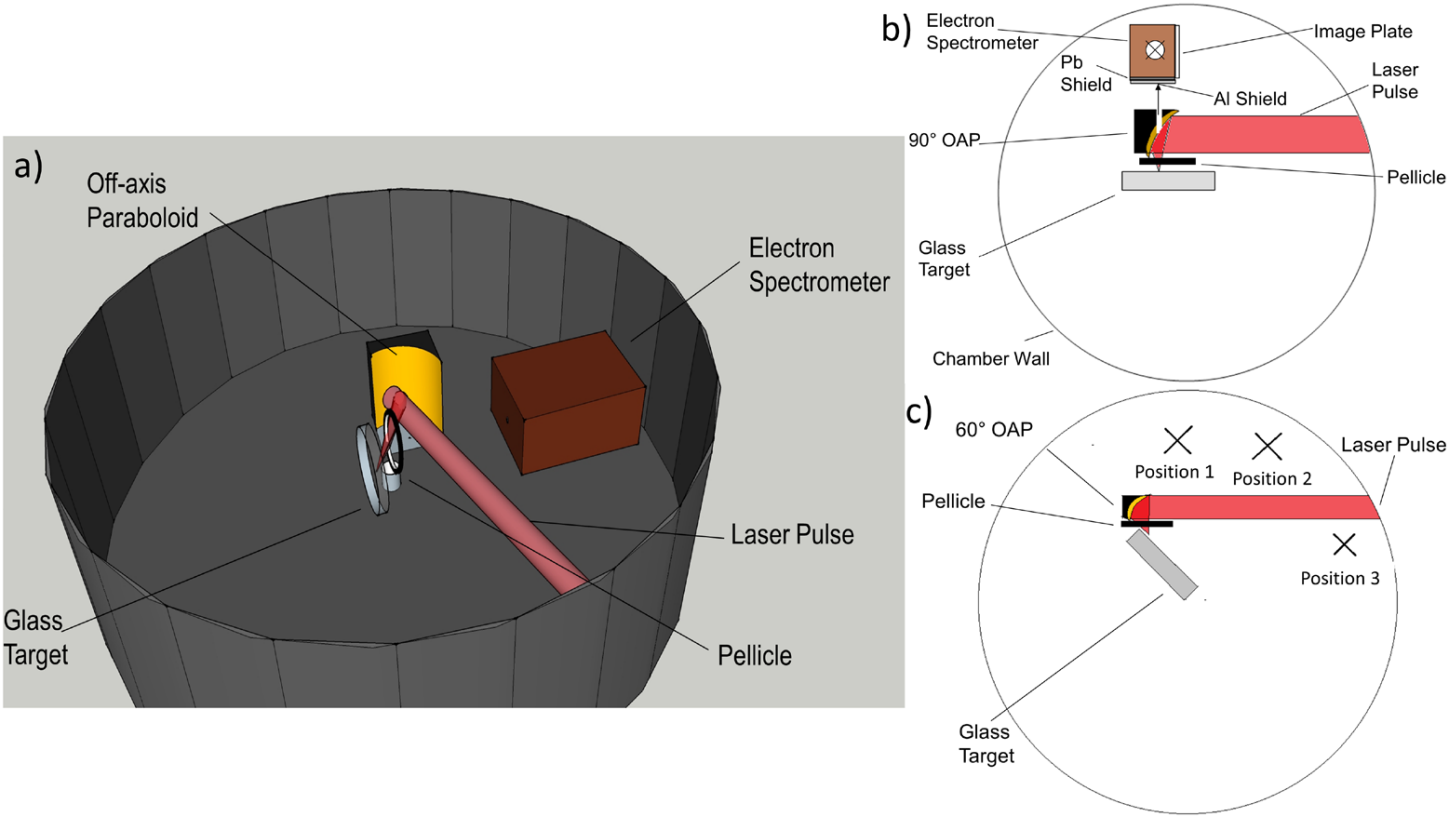
Schematic of the experimental setup (**a**) and top view of the normal incidence geometry (**b**) as well as the grazing incidence geometry (**c**). Electron spectrometer: 1.15 kG magnetic spectrometer with a Fujifilm MS image plate covered with lead and aluminum shielding; pellicle: 2-inch diameter, 2 $\mu m$ thick nitrocellulose pellicle; target: 4-inch diameter, 6 mm thick glass; OAP: off-axis paraboloid. The spectrometer is placed at three different positions in (**c**).

The experiments were performed at the University of Michigan, using the $\lambda ^3$ laser system with 30 fs, 12 mJ, $0.8\,\upmu m$ pulses at a repetition rate of 480 Hz. The laser contrast is $\sim 10^8$ within a picosecond. The experimental setup is shown in Fig. 8. A P-polarized laser pulse was focused from a $90^{\circ }$ ($60^{\circ }$) f/1 gold off-axis paraboloid onto the target at normal (grazing) incidence. Note that a hole was drilled in the center of the $90^{\circ }$ OAP in order to capture signals on the spectrometer. The geometry of normal and grazing incidence cases are drawn in Fig. 8b,c, respectively. Note that in such tight focus setup the rays cover a range of angles (approximately $\pm 20^{\circ }$). The angle of the central ray in grazing incidence is estimated to be $\sim 70^{\circ }$, which is as close to grazing as the beam could get without clipping. The focal spot size (FWHM) was $1.5\,\upmu \text {m}$, resulting in a peak intensity $I=\frac{0.88\cdot E_p}{\tau _p\cdot \pi \cdot w^2/2}=1.5\times 10^{19}\;\text {W}/\text {cm}^2$ and normalized vector potential $a_0\sim$ 2.5. The laser was focused through a thin pellicle to protect the OAP from debris. A rotary target stage was used to provide degrees of freedom in rotation as well as movement radially and longitudinally. Most of the experiments were performed with glass targets unless specified to be copper. An external prepulse was available by placing a thin pellicle in the optical system to pick up $\sim 8\%$ of the pulse energy. The prepulse intensity is around $5\times 10^{16}\text {W}/\text {cm}^{2}$. The generated electrons were diagnosed by a Fuji MS image plate after deflected off a 1.15 KG magnetic field. The image plate efficiency was included using the published calibration data^26^. Lead and aluminum shieldings were used to prevent noise from Bremsstrahlung radiation. Detailed description of the spectrometer can be found in reference^12,13^.

PIC simulations were performed using the OSIRIS^43,44^ 4.4.4 framework in 2D3v Cartesian geometry. There are 100 macroparticles per cell and the grid size is $\lambda /32\times \lambda /32=0.025 \,\upmu \text {m}\times 0.025 \,\upmu \text {m}$, where $\lambda =0.8 \,\upmu \text {m}$ is the laser wavelength. The convergence of the simulation is checked using up to $\lambda /64\times \lambda /64$ grid size. The computational time-resolution is 0.027 fs or $\sim 100$ step per laser cycle. The laser pulse is assumed to be Gaussian in both the longitudinal and transverse direction with a FWHM pulse duration of $\tau =30 fs$. The laser pulse is continuously launched from the wall and focused down to beam waist $w0=fwhm\times 1.699/2=1.28\,\upmu \text { m}$, $a_0=2.5$. The simulation box size is $64 \,\upmu \text {m}\times 64\,\upmu \text { m}$ in normal ($0^{\circ }$) incidence geometry, $48 \,\upmu \text {m}\times 48 \upmu \text {m}$ in oblique ($45^{\circ }$) incidence geometry and $72 \,\upmu \text {m}\times 28\,\upmu \text {m}$ in grazing ($76^{\circ }$) incidence geometry. The reported angles are those of the central rays. The simulations were run for 270 fs, 210 fs, and 300 fs, respectively, utill the interesting electrons left the simulation box. The initial plasma density profile is described by a uniform glass-solid-density ($5.01 \times 10^{22}\,{\text {cm}}^{-3}$) region plus an exponential tail, as is illustrated in Fig. 1f.

## Supplementary information

Supplementary Video 1.

Supplementary Video 2.

Supplementary figure.
