# Supplementary material for: Towards isolated attosecond electron bunches using ultrashort-pulse laser-solid interactions
Source: Sci Rep. 2020 Oct 27;10:18354. doi: 10.1038/s41598-020-75418-6 (PMC7591899; doi:10.1038/s41598-020-75418-6)
Supplement: Supplementary file 3 — Supplementary figure. [file 41598_2020_75418_MOESM3_ESM.docx]

Supplementary Information

**Towards isolated attosecond electron bunches using ultrashort-pulse laser-solid interactions**

Jinpu Lin, Thomas Batson, John Nees, Alexander G.R. Thomas, and Karl Krushelnick

Center for Ultrafast Optical Science, University of Michigan, Ann Arbor, MI 48109, USA

*Supplementary Fig. S1* is a sketch of the focal spot geometry mentioned on page 5 in the manuscript.


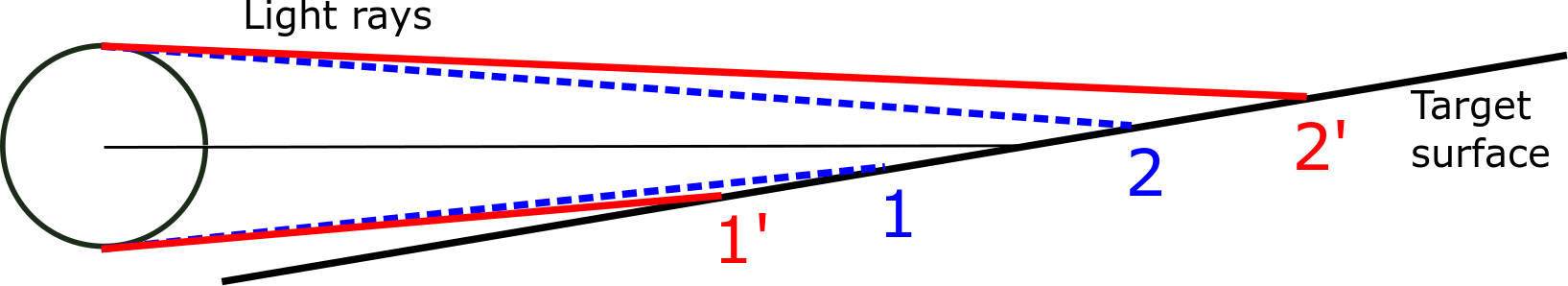


Supplementary Fig. S1. Incidence angle increases with focal spot

*Supplementary Fig. S2* is an example of the raw data from the image plate.


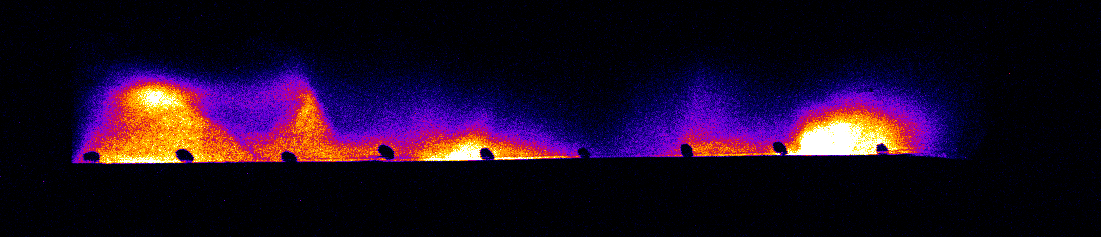


Supplementary Fig. S2. Raw data from the image plate.

A video recording energetic electrons in the simulation box is attached separately as *supplementaryVideoS1.mp4*. It is mentioned on page 6 in the manuscript.

A video recording electron bunches from a tilted laser pulse is attached separately as *supplementaryVideoS2.mp4*. It is mentioned on page 7 in the manuscript.
